# 325. Empiric Antibiotics for COVID-19 and the Utility of Procalcitonin

**DOI:** 10.1093/ofid/ofab466.527

**Published:** 2021-12-04

**Authors:** Adrienne D Desens, Kiya D Mohadjer, Jessica Thompson

**Affiliations:** 1 Renown Regional Medical Center, Reno, Nevada; 2 Renown Health, Reno, Nevada

## Abstract

**Background:**

Bacterial coinfection in COVID-19 is infrequent, yet empiric antibiotic use is common. The objectives of this study were to investigate the effect of empiric antibiotics on time to resolution of COVID-19 pneumonia, elucidate the impact of COVID-19 on procalcitonin levels, and determine the incidence of respiratory bacterial coinfection.

**Methods:**

This was a retrospective study of adult patients hospitalized with COVID-19 between June 1, 2020 and September 30, 2020. Patients were included if they had at least one procalcitonin level. They were excluded if admitted to an intensive care unit within 24 hours of presentation or received antibiotics for an indication besides pneumonia. Patients were stratified into 4 groups based on procalcitonin level and receipt of antibiotics. The primary outcome was time to clinical resolution of pneumonia. A key secondary outcome was incidence of confirmed respiratory bacterial coinfection.

**Results:**

A total of 199 patients were included. Patients with a procalcitonin greater than 0.25 ng/mL who received antibiotics had a longer median time to clinical resolution of pneumonia, 8 days (95% CI, 4 to 11 days) vs. 3 or 4 days in other groups (P< 0.001). Additionally, this same group required greater baseline oxygen supplementation, had more comorbidities, and increased mortality compared to all other groups. Median time to clinical resolution of pneumonia was also longer in patients who received antibiotics compared to those who did not (5 vs. 4 days, P=0.017) and in those with a procalcitonin greater than 0.25 ng/mL compared to those with PCT less than or equal to 0.25 ng/mL (7 vs. 4 days, P< 0.001). Renal dysfunction was more prevalent in patients with an elevated procalcitonin (45% vs. 17.5%). The overall incidence of confirmed respiratory bacterial coinfection was 1.5%.

**Conclusion:**

Irrespective of procalcitonin level, empiric antibiotics were not associated with a shorter time to resolution of COVID-19 pneumonia in non-critically ill patients. Elevated procalcitonin is likely a reflection of the severity of COVID-19 disease and baseline renal function rather than bacterial infection. Additionally, the overall incidence of confirmed bacterial coinfection in non-critically ill patients hospitalized with COVID-19 was low.

**Disclosures:**

**Kiya D. Mohadjer, PharmD, BCPS, BCIDP**, **Eli Lilly and Company** (Shareholder)**Gilead Sciences** (Shareholder)

